# Disseminated Cryptococcal Disease in a Patient With Monoclonal Gammopathy of Undetermined Significance and Polycythemia Vera: A Case Report and Review of the Literature

**DOI:** 10.7759/cureus.12458

**Published:** 2021-01-03

**Authors:** Muhammad Khurram Guhjjar, Haider Ghazanfar, Shoaib Ashraf, Maneesh Gaddam, Ajsza Matela

**Affiliations:** 1 Internal Medicine, Bronxcare Health System, Bronx, USA; 2 Internal Medicine, Bronx Care Health System, Icahn School of Medicine at Mount Sinai, Bronx, USA; 3 Pulmonary and Critical Care Medicine, Bronxcare Health System, Bronx, USA

**Keywords:** cryptococcoma, monoclonal gammopathy of undetermined significance, liposomal amphotericin b, polycythemia vera, immunocompetent, cryptococcal meningitis

## Abstract

Cryptococcosis is a life-threatening opportunistic infection caused by Cryptococcus gattii and Cryptococcus neoformans. It affects both immunocompetent and immunosuppressed hosts. Disseminated cryptococcal infection is rare in immunocompetent patients, but the cryptococcal disease's neurological sequelae may be more prominent in this group. We present a case of a 58-year-old male patient with medical comorbidities of monoclonal gammopathy of undetermined significance (MGUS) and polycythemia vera. The patient presented with gradual worsening of mental status over one week. He was found to have Cryptococcus neoformans meningoencephalitis and fungemia. The patient received two weeks of liposomal amphotericin B (LAmB) and flucytosine with excellent clinical response. He was discharged on high dose fluconazole, and he returned to the hospital in one week with new-onset hemiplegia and cryptococcomas on imaging. Prolonged intravenous (IV) treatment of six weeks duration resulted in significant clinical improvement and disease-free state at two years follow-up. This article aims to stress the importance of individualized prolonged IV treatment with liposomal amphotericin B and flucytosine despite good initial response in patients with polycythemia vera and MGUS. This is the first reported case of cryptococcal disease, to the best of our knowledge, in a patient with MGUS and the third case of cryptococcal infection in patients with polycythemia vera in a non-HIV non-transplant state. Prolonged individualized IV treatment should be considered in immunocompetent patients with the above conditions, as this condition, if not adequately treated and relapses, lead to high morbidity and mortality.

## Introduction

Cryptococcal disease (CD) refers to an infection by the genus cryptococcus [1]. There are two common pathogenic species in humans, including Cryptococcus neoformans and Cryptococcus gatti [2]. The incidence of the cryptococcal disease has increased during the era of human immunodeficiency (HIV) [3]. The cryptococcal disease has been increasingly reported in HIV-negative patients and immunosuppression states such as solid organ transplantation, hematologic malignancy, glucocorticoid use for a prolonged time, and chronic liver disease, and sarcoidosis [4-5]. This is particularly important as the cryptococcal disease's neurological sequelae may be more prominent in non-HIV patients [6-7]. Significantly higher mortality rates are observed in HIV-negative patients with the cryptococcal disease. Cryptococcus gatti has been shown to have a predilection for immunocompetent hosts [8-9]. Pappas et al. conducted a multicenter case series involving 306 patients in the United States, which concluded that up to 20% of cryptococcosis cases had no significant predisposing medical condition or immunosuppression state [10]. Recent data suggest that CD in HIV-negative patients could be a complication of an underlying undiagnosed secondary immunodeficiency [11]. We report a case of Cryptococcus neoformans meningoencephalitis and fungemia in a patient with monoclonal gammopathy of undetermined significance (MGUS) and polycythemia vera on hydroxyurea. This article aims to highlight the rare cause of cryptococcal disease in immunocompetent patients and consideration of individualized prolonged intravenous (IV) treatment in patients with MGUS and polycythemia vera as there might be an association of nonmalignant myeloproliferative syndromes and cryptococcal infection. Cryptococcal disease, if not adequately treated, is associated with higher morbidity and mortality rate.

## Case presentation

**Figure 1:**
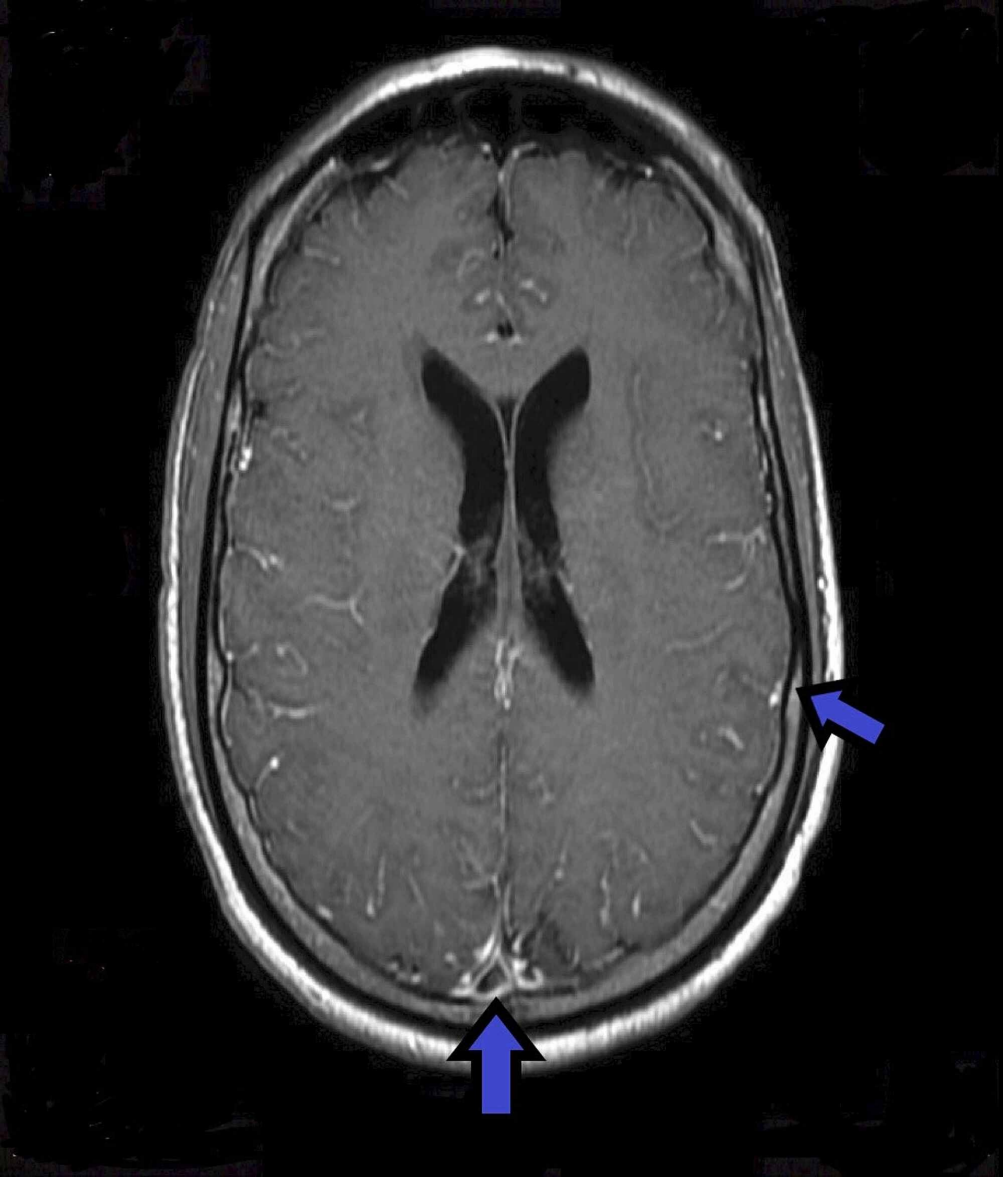
Magnetic resonance images of the brain with and without contrast showing meningeal enhancement but no basal ganglia lesion Blue arrows indicate meningeal enhancement.

**Figure 2:**
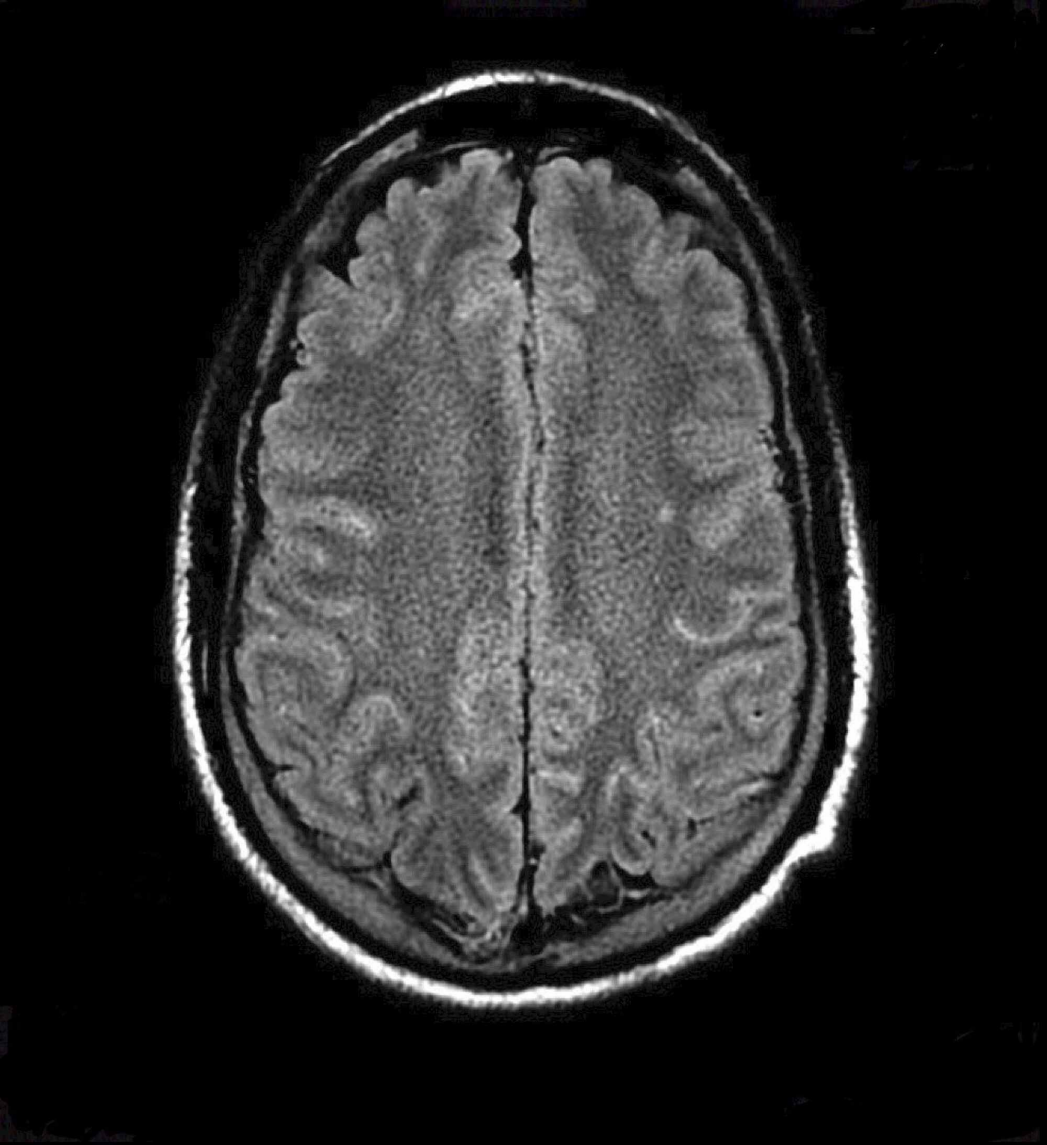
Magnetic resonance images of the brain with fluid-attenuated inversion recovery without any basal ganglia lesion

Our patient is a 58-year-old HIV-negative African American male with medical comorbidities significant for Janus kinase 2 (JAK) positive polycythemia vera, MGUS, and hypertension presented with gradual worsening of mental status for one week. The patient's family reported intermittent confusion, drowsiness, and auditory hallucinations. He had no history of substance use, high-risk sexual behavior, or recent travel. In the emergency department, the patient was found to have a blood pressure of 122/69 millimeters of mercury, heart rate of 92 beats/minute, respiratory rate of 16/minute, saturating 99% on room air, and temperature of 36.8° C. On physical examination, the patient was oriented to person only and was inconsistently following commands. Pupils were equal and reactive to light bilaterally. There was no nuchal rigidity. Kernig's and Brudzinski's signs were negative. The patient was moving all extremities. The remaining central nervous system examination was limited due to the patient's confused state. Laboratory results showed hemoglobin of 16.3 g/dl, platelets of 720 k/ul, and white blood cell count of 48.1 k/ul with 95.5% neutrophils. This complete blood count was unchanged from his baseline chronic leukocytosis and thrombocytosis. Other laboratory results, including liver function tests, thyroid function tests, vitamin B12 level, rapid plasma reagin (RPR), and urinalysis, were within normal limits. Urine toxicology screen was negative for any substance use. Chest radiography was normal. Head computed tomography (CT) non-contrast showed no acute cerebrovascular accident. Brain magnetic resonance imaging (MRI) with and without contrast revealed meningeal enhancement suggestive of infective versus inflammatory changes. This has been shown in Figures 1-2.

On day 2 of hospitalization, the patient developed a fever of 38.4° C (101.2 F). Antibiotic therapy with ceftriaxone, vancomycin, and acyclovir was initiated for empiric treatment of meningitis and encephalitis. A lumbar puncture was performed. The cerebrospinal fluid (CSF) analysis showed high opening pressure and high protein, lactic acid, and white cell count, as presented in Table 1.

**Table 1:** Cerebrospinal fluid analysis on the first presentation

Cerebrospinal fluid	Cerebrospinal fluid analysis	Normal values
Opening pressure	55 cmH2O	10-20 cmH2O
Appearance	Colorless	Colorless
Protein	113 mg/dL	15-45 mg/dL
Glucose	13 mg/dL	40-70 mg/dL
Lactic acid	6.7 mmoles/L	0.6- 2.2 mmoles/L
White blood cell count	189	<5
Neutrophil count	12%	
Lymphocytes	84%	
Red cell count	4	<10
Cryptococcal Ag titer	1:1024	

Yeast was found in CSF staining. Cryptococcal antigen was positive on CSF analysis. The patient was found to have positive serum cryptococcal antigen (titer 1:1024). He was later identified to have Cryptococcus neoformans on CSF final culture report. Admission blood culture also grew Cryptococcus neoformans. Liposomal amphotericin B and flucytosine were started. CSF Mycobacterium tuberculosis polymerase chain reaction (PCR), urine Histoplasma antigen, cytomegalovirus (CMV), varicella-zoster virus, herpes simplex virus and John Cunningham (JC) virus PCR were all negative. He was tested for HIV infection using a 4th generation immunoassay and HIV viral load measurement with a quantitative PCR assay - both were negative. HIV-1 viral load was <20 IU/ml. His cluster of differentiation 4 (CD4) T lymphocytes were above 450/MCL on two different occasions (two weeks apart). Anti-nuclear antigen was negative. Immunofixation electrophoresis (IFE) revealed a mild monoclonal immunoglobulin M (IgM) spike. Subsequently, the patient underwent a bone marrow biopsy, which demonstrated hypercellular marrow with trilineage maturation and 3% mature plasma cells. Features were consistent with a myeloproliferative disorder. Hematological workup showed no evidence of acute leukemia or multiple myeloma.

The patient continued to improve throughout treatment, and his mental and functional status returned to baseline. Repeat lumbar puncture had opening pressure of 17 cmH2O. Cryptococcal antigen titer in serum dropped to 1:28. After completing 14 days of induction therapy with IV liposomal amphotericin B and flucytosine, he was discharged home on consolidation therapy of fluconazole 800 milligrams daily for eight weeks to be followed by 200 milligrams of fluconazole daily for at least one year.

One week after discharge, the patient returned to the emergency department with a new-onset of left-sided weakness despite reported adherence to fluconazole. The patient was drowsy but fully oriented to time, place, and person. Physical examination revealed a left flat nasolabial fold, muscle strength of 0/5 in the left lower extremity, and 3/5 in the left upper extremity. Repeat head CT scan without contrast was negative for acute bleed but revealed mild ventricular dilatation suspicious for communicating hydrocephalus. Repeat lumbar punctures (LP) was performed with an opening pressure of 19 cm H2O and CSF sample positive for cryptococcal antigen (titer 1: 1024). Serum Cryptococcal titer was 1:1024. CSF analysis is presented in Table 2.

**Table 2:** Cerebrospinal fluid analysis on the second presentation

Cerebrospinal fluid	Cerebrospinal fluid analysis	Normal values
Opening pressure	19 cmH2O	10-20 cmH2O
Appearance	Colorless	Colorless
Protein	177 mg/dl	15-45 mg/dL
Glucose	18 mg/dl	40-70 mg/dL
Lactic acid	2 mmoles/L	0.6- 2.2 mmoles/L
White blood cell count	38	<5
Neutrophil count	17%	
Lymphocytes	78%	
Red cell count	0	<10
Cryptococcal Ag titer	1:1024	

**Figure 3:**
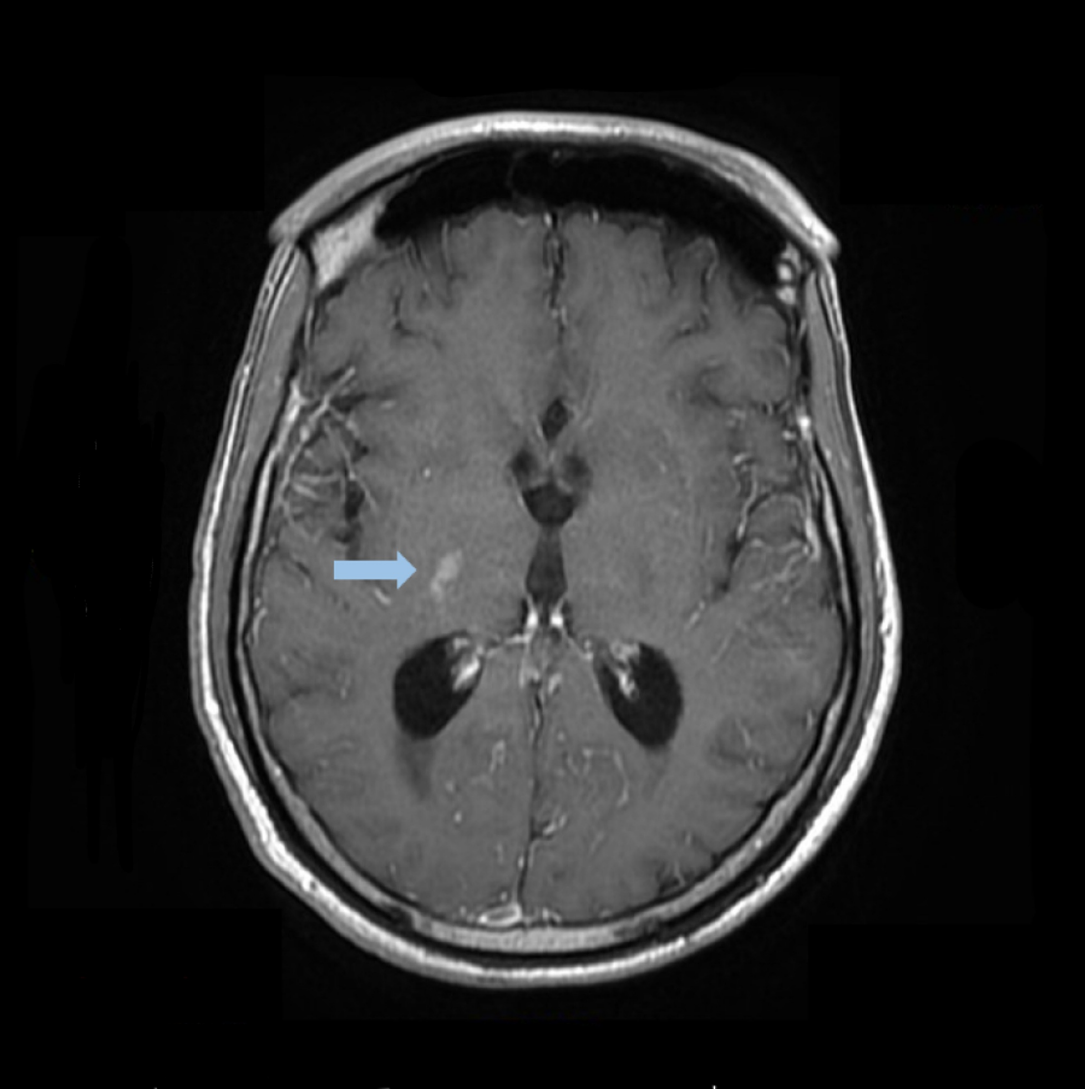
Magnetic resonance images of the head with contrast showing right basal ganglia lesion Blue arrow indicates cryptococcomas lesion.

**Figure 4:**
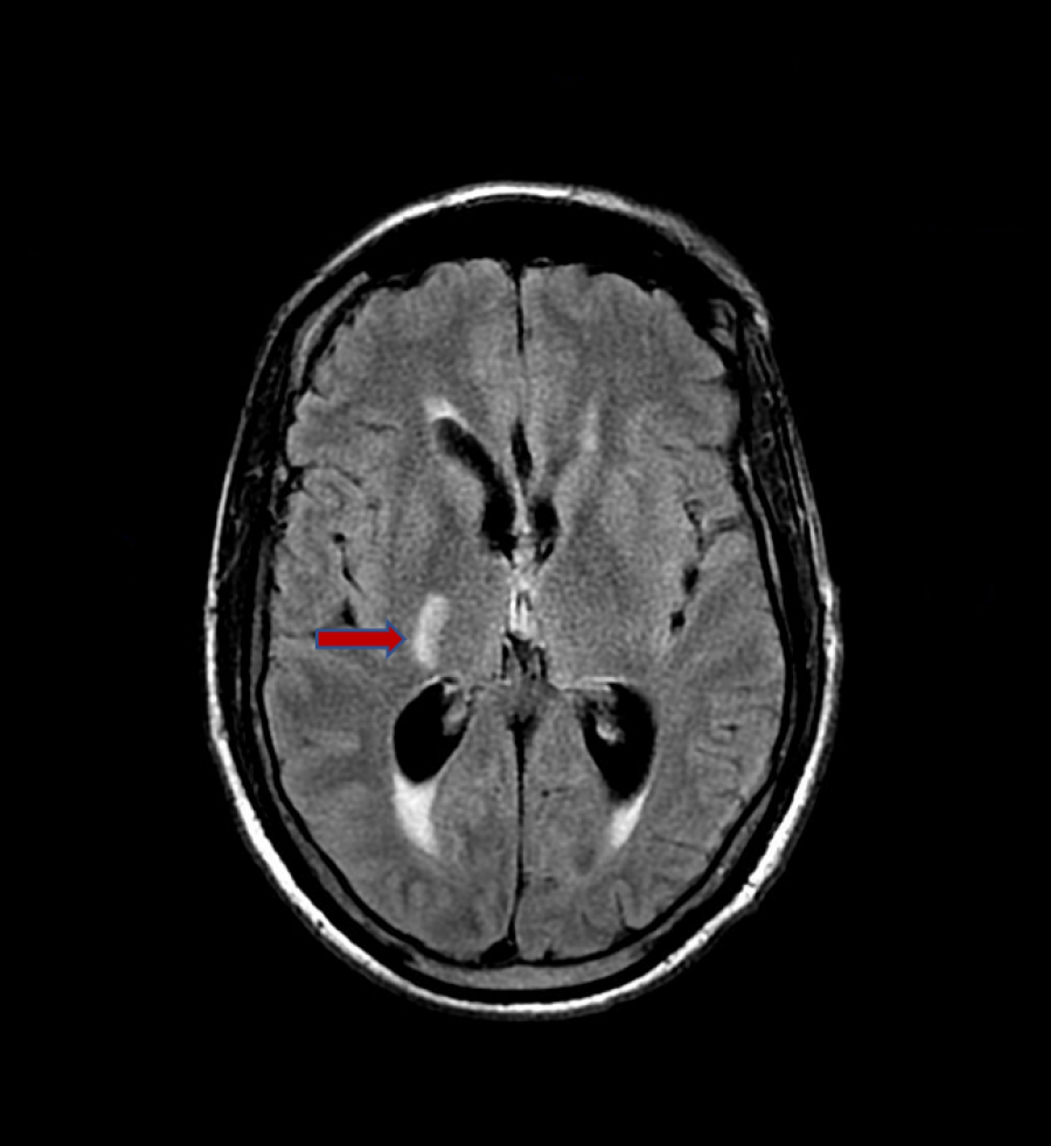
Magnetic resonance images of the brain with fluid-attenuated inversion recovery showing right basal ganglia lesion Red arrow indicates cryptococcomas lesion.

Blood culture remained negative for any fungal element growth. Brain MRI with contrast revealed a restricted diffusion area in the right basal ganglia and 0.8 x 0.5-centimeter lesion in the genu of the right corpus callosum suggestive of cryptococcoma without surrounding edema or mass effect. This has been shown in Figures 3-4.

Magnetic resonance angiography and venography of the brain were negative for occlusion, aneurysm, or thrombosis. The patient was transferred to the intensive care unit (ICU), and IV liposomal amphotericin B and flucytosine were reinstituted. His left-sided weakness improved throughout treatment, with 3/5 in the left lower extremity and 4/5 in the left upper extremity. He completed six weeks of inpatient IV L-AmpB plus flucytosine with remarkable improvement in his clinical status, and serum cryptococcal titer dropped to 1:16 and then to 1:8. He was then discharged to short term rehabilitation center on maintenance therapy with fluconazole and was later discharged home. To date, at two years of follow-up in the clinic, the patient has been maintained on oral fluconazole therapy, has shown no relapse, and has been actively living without any deficits.

## Discussion

Cryptococcus neoformans meningoencephalitis is an invasive and most encountered manifestation of cryptococcal disease. The pathogenic cryptococcus neoformans is commonly classified into three varieties: Cryptococcus neoformans variant grubii (serotype A), variant Gatti (serotypes B and C), and Cryptococcus neoformans variant neoformans (serotype D). Serotype A is responsible for 95% of infection and is frequently isolated, but serotype D is more common in Europe. Distribution of cryptococcus gattii is mainly in Papua new guinea, Australia, and less commonly in Canada, sub-Saharan Africa, and the United States northwest [12]. Cryptococcus neoformans causes infection via inhalation of spore or yeasts form of the fungus from bird excreta, especially pigeons and chickens, through the respiratory tract. Thus, the lungs constitute the entry point for the cryptococcus neoformans infection. After the fungus enters the body, it can spread to several organs, including the brain, meninges, liver, gut, kidneys, pancreas, and spleen [13]. Whether cryptococcus neoformans infection causes focal pneumonitis and disseminated cryptococcal disease depends upon the virulence of the fungal strain, the burden of the exposure, and the patients' immunological status. Depending on the immunological status, the infection can cross the blood-brain barrier through fungal neurotropism and cause neurological impairment. Most patients with central nervous system (CNS) cryptococcus disease usually manifest as meningoencephalitis, meningitis, encephalitis, or ventriculitis, presenting with signs and symptoms of headache, fever, personality changes, lethargy, and memory loss.

Patients with polycythemia vera are possibly at higher risk for cryptococcosis as two cases were reported in the literature with cryptococcal meningitis and cutaneous cryptococcosis [14, 15]. We searched for articles in PubMed and Google Scholar databases till May 20th, 2020, with the following keywords: "monoclonal gammopathy of undetermined significance (MGUS)", "polycythemia vera", "Cryptococcus", "AND" for a possible association of MGUS and cryptococcal disease. To our best knowledge, this is the first case report of cryptococcal disease in a patient with MGUS and the third case report in patients with preexisting polycythemia vera. It is, perhaps, essential to look for these conditions in patients with CD as there might be an association of nonmalignant myeloproliferative syndromes and cryptococcal infection.

CD in the non-HIV, non-transplant (NHNT) population is associated with poorer outcomes, including increased morbidity and mortality [4, 5]. CNS involvement is more prominent in the NHNT papulation with higher morbidity. In rare cases, the chronic granulomatous process can lead to the formation of a cryptococcoma with a tumor-like appearance, which leads to the speculation that a more intact immune system is responsible for higher morbidity in the immunocompetent group [6, 7]. Similarly, a robust immune system could be the reason that cryptococcomas are more likely to be present in immunocompetent patients than in immunosuppressed patients.

Diagnosis of CD can be made with serum and CSF antigen testing with imaging studies. Serum cryptococcal polysaccharide antigen test is very accurate with sensitivity and specificity of 100% and 96-99.5%, respectively. CSF antigen test has sensitivity and specificity of 96-100% and 93.5-99.8%, respectively [16]. On MRI, intraparenchymal cryptococcosis can present with some hypointense areas on Tl-weighted image (T1WI) and high intensity or hyperintense areas on T2-weighted image (T2WI).

Guidelines for cryptococcal disease management were published in 2010 by the Infectious Diseases Society of America (IDSA), which recommended that the NHNT population get amphotericin B and flucytosine for four weeks as induction treatment. But patients with neurological sequelae, induction therapy should be extended to a minimum of six weeks, followed by consolidation and maintenance treatment with fluconazole. In immunocompetent patients without risk of treatment failure and excellent clinical response, induction therapy can be reduced to two weeks [17]. IDSA recommendations for cerebral cryptococcomas include induction therapy with liposomal amphotericin B and flucytosine for six weeks, followed by consolidation and maintenance treatment with fluconazole for six to eighteen months. Patients with cerebral cryptococcomas with surrounding edema and mass effect should also be on adjunctive therapy with corticosteroids [17]. It is essential to keep patients on prolonged maintenance therapy to avoid relapse, especially in the first year post-therapy [18]. Large cerebral cryptococcomas (≥3 cm) should be treated with open surgical excision or stereotactic-guided debulking [17]. 

## Conclusions

We recommend individualizing treatment duration for disseminated Cryptococcus infection in immunocompetent patients with MGUS and polycythemia vera as there might be an association of nonmalignant myeloproliferative syndromes and cryptococcal infection. Given the high morbidity and mortality associated with this disease, extending induction therapy to six weeks should be considered despite initial clinical improvement. 
